# The Glycocalyx Shedding Influences Hemodynamic and Metabolic Response to Fluid Load in Septic Shock

**DOI:** 10.5152/TJAR.2021.21224

**Published:** 2022-04-01

**Authors:** Yana Ilyina, Eugenia Fot, Vsevolod Kuzkov, Mikhail Kirov

**Affiliations:** 1 Department of Anaesthesiology and Intensive Care, Northern State Medical University, Arkhangelsk, Russian Federation; 2 Department of Anesthesiology and Intensive Care, City Hospital # 1, Arkhangelsk, Russian Federation

**Keywords:** Endothelial glycocalyx, hemodynamics, heparan sulfate proteoglycan, septic shock, syndecan 1

## Abstract

**Objective:**

Sepsis-associated endothelial dysfunction and degradation result in release of inflammatory mediators, compromise endothelial permeability, and impair alveolar fluid clearance leading to pulmonary edema. Excessive fluid therapy in septic shock damage the endothelial glycocalyx which will increase capillary leakage. The aim of our study was to assess the relationship of endothelial glycocalyx shedding with hemodynamic and metabolic response to fluid load in patients with septic shock.

**Methods:**

Eighteen adult patients were included in prospective observational study. To predict the response to infusion, we performed fluid load test by using crystalloids 7 mL kg^−1^ for 10 minutes. The plasma concentrations of endothelial glycocalyx components including heparan sulfate proteoglycan and syndecan 1 were measured at baseline, 2, 24 hours after fluid load test.

**Results:**

We observed associations of syndecan 1 with extravascular lung water index (*rho *= 0.48, *P*  = .04) at baseline and of heparan sulfate proteoglycan with extravascular lung water index (*rho* = −0.56, *P *= .03) and pulse pressure variation (*rho *= 0.53, *P  *= .04) at 24 hours after fluid load test. The plasma concentration of syndecan 1 correlated with lactate at baseline (*rho *= 0.51, *P *= .02) and at 24 hours after fluid load test (*rho *= 0.76, *P *= .009). At 2 hours after fluid load test, the concentration of syndecan 1 correlated with global end-diastolic volume index (*rho*= 0.93, *P *= .001) in normovolemic patients.

**Conclusions:**

The shedding of endothelial glycocalyx after fluid load test in septic shock is associated with hemodynamic and metabolic responses and related with the severity of pulmonary edema.

**Main Points**

- The response of endothelial glycocalyx to sepsis depends on fluid therapy with increased glycocalyx shedding after achieving normal volume status of the patient.
- In septic shock, the concentration of endothelial glycocalyx components correlates with parameters of preload, severity of pulmonary edema, and plasma lactate.
- The accumulation of extravascular lung water associates with plasma concentration of syndecan 1 during early phase of septic shock, and with heparan sulfate proteoglycan at 24 hours after the fluid load.

## Introduction

In patients with septic shock, fluid therapy is one of the key “golden hour” interventions. However, it can be accompanied by several potentially dangerous side effects, especially in patients with cardiac comorbidities, acute respiratory distress syndrome (ARDS), and severe “capillary leak”.^1-4^ Thus, before a decision regarding infusion, it is important to determine whether this patient will respond to fluid load with an adequate increase in cardiac output or stroke volume, usually by between 10% and 15% of the initial value.^5^ Moreover, even in the case of fluid responsiveness, increased permeability can lead to progressive tissue edema and organ dysfunction.^6^

Nowadays, the effects of fluids on sepsis-induced degradation and shedding of endothelial glycocalyx (EG) and, thereafter, the capillary leakage resulting in the development of interstitial edema are being actively discussed.^7-11^ Indeed, glycocalyx plays a key role in the physiology of microcirculation and endothelium integrity and is involved in the regulation of microcirculatory tone and vascular permeability, maintaining the oncotic gradient across the endothelial barrier, leukocyte adhesion, and migration as well as preventing the thrombosis.^12,13^ Shedding and flaking of EG result in an instant increase in free plasma concentrations of syndecan 1 (S1) and heparan sulfate proteoglycan (HSPG), which can be determined by enzyme-linked immunosorbent assay.^14^

Sepsis and septic shock are associated with severe involvement of the endothelium and EG degradation that leads to dysregulation of homeostasis and permeability of the vascular wall causing damage to the microvasculature.^7,15,16^ In sepsis, the damaged EG layer becomes thinner resulting in extravasation of proteins and fluid into the interstitial space and causing hypovolemia, hypoalbuminemia, overhydration, and tissue edema.^16^ Thus, critical remodeling of the endothelial system and EG triggers the mechanism of multiple organ failure. However, the relationship between EG and fluid therapy of sepsis is still a subject of debate.

The aim of our study was to assess the association of free plasma fraction of key EG components with hemodynamic and metabolic response to fluid therapy, as well as severity of pulmonary edema, in patients with septic shock.

## Methods

The prospective observational study was approved by the Research Ethical Committee (No. 02/06-16) of the Northern State Medical University (Arkhangelsk, Russian Federation). Written informed consent was obtained from the patient or next of kin if the patient was unconscious. Twenty-one adult patients with the diagnosis of septic shock were screened for the study entry. Three patients were excluded (2 patients due to progressing refractory shock and death during the screening period and 1 patient due to dysfunction of the thermodilution catheter), thus totally 18 patients were enrolled and underwent further analysis. The inclusion criteria were the presence of informed consent, the diagnosis of septic shock, the age of patients > 18 years, the allocation at the intensive care unit (ICU), and the requirement of mechanical ventilation. All patients were sedated and received neuromuscular blockers during fluid load test. The exclusion criteria were right heart failure, arrhythmias, and abdominal compartment syndrome. 

Sepsis and septic shock were diagnosed using Sepsis-3 criteria. During the study, the patients received the therapy according to the guidelines of Surviving Sepsis Campaign.^17^ The doses of vasopressors were titrated to maintain mean arterial pressure within 65-85 mm Hg.

### Hemodynamics

In all patients, we catheterized the internal jugular or subclavian vein with a triple-lumen central venous catheter (Certofix, B|Braun, Germany) and the femoral artery with a thermistor-tipped arterial catheter (5F, PV2015L20, Pulsion Medical Systems, Munich, Germany). The arterial blood pressure was recorded from a side port of the catheter. Hemodynamic monitoring was carried out using the method of transpulmonary thermodilution (PiCCO_2_ monitor, Pulsion Medical Systems) by a triplicate 15 mL bolus of cold (<8°C) 0.9% saline solution. Cardiac index (CI), global end-diastolic volume index (GEDVI), extravascular lung water index (EVLWI), pulmonary vascular permeability index (PVPI), central venous pressure (CVP), systemic vascular resistance index (SVRI), pulse pressure variation (PPV), and stroke volume variation (SVV) were assessed using transpulmonary thermodilution and arterial pulse contour analysis.

### Fluid Load Test

The fluid bolus was administered within 24 hours from the onset of shock. Fluid load test (FLT) was performed by the intravenous infusion of 7 mL kg^−1^ of an isotonic balanced solution (Sterofundin-Iso, B|Braun, Germany) within 10 minutes. The responsiveness to fluid load was defined as an increase in cardiac output after FLT by ≥15% from the initial values, which allowed us to divide the patient data into groups of responders or nonresponders.

### Components of Endothelial Glycocalyx

In all patients, blood samples were taken at study baseline (before FLT), and at 2 and 24 hours after FLT. Immediately after sampling, blood was centrifuged at 2000 g for 10 minutes. The plasma fraction was immediately frozen and stored at −80°C. The components of EG including HSPG and S1 were assessed by using an enzyme-linked immunosorbent assay kit (ELISA Kits for HSPG, SDC 1, Sugar Land, TX, United States USA).

### Other Measurements

In addition to hemodynamics and components of EG, we assessed blood gases (ABL Flex 800 Radiometer, Denmark), biochemical parameters (Random Access A-25, BioSystems, Barcelona, Spain), doses of vasopressors, Sequental Organ Failure Assessment (SOFA) scores, duration of ICU and hospital stay, and mortality at day 28. The measurements of hemodynamics, biochemistry, and blood gases were performed at baseline, immediately after FLT and at 2 and 24 hours thereafter. 

### Statistical Analysis

For data collection and analysis, we used the Statistical Package for Social Sciences, version 17.0 software (SPSS Inc.; Chicago, IL, USA). and MedCalc software (version 12.3, MedCalc Software, Belgium). Data distribution was assessed using the Shapiro–Wilk test. Data were presented as mean ± standard deviation (SD) or median (25th-75th percentile). The comparisons with baseline were performed using the Wilcoxon rank test. Nominal data were compared using χ^2^ test or Fisher exact test and expressed as patient number. For correlation analysis, we used Spearman’s *rho*. *P* values < .05 were regarded as statistically significant.

## Results

The baseline clinical characteristics of study patients are presented in Table 1. All patients had severe septic shock requiring maintenance of hemodynamics by fluid resuscitation with positive fluid balance and high doses of vasopressors. The sources of sepsis included abdominal (67%) and respiratory (33%) infections. The survival rate at day 28 was 56%.

The hemodynamic and metabolic variables as well as blood gases of study patients are summarized in Table 2. Cardiac index and PVPI increased by 17% and 30% from baseline in 24 hours, respectively (*P* < .05). Other parameters did not change significantly.

Ten patients (56%) were considered as fluid responders. We did not find significant differences in plasma fraction of S1 and HSPG, as well as in hemodynamics and clinical characteristics, between the groups of responders and non-responders at all study stages. The intragroup changes in study parameters compared to baseline in responders and nonresponders are shown in Table 3. At 2 hours after FLT, we found a significant decrease in heart rate in responders (*P*  = .01). Also, in the group of responders, there were increments in GEDVI at 2 hours after FLT and in EVLWI at 24 hours after FLT (*P* < .01). In addition, at 24 hours, the requirement of norepinephrine reduced in the responder group (*P*  = .01). At 2 hours after FLT, the nonresponder group demonstrated the decrease in PPV from 16% to 8% (*P*  = .03). At 24 hours, both mean arterial pressure and SVRI decreased whereas CI rose compared with baseline in nonresponders (*P* < .05). The duration of ICU and hospital stay as well as mortality did not differ significantly between responders and nonresponders.

Based on the GEDVI value, we identified the groups of patients with hypovolemia (GEDVI < 650 mL m^−2^, n = 7) and normovolemia (GEDVI 650-850 mL m^−2^, n = 11). At 2 hours after FLT, concentration of S1 in the normovolemic group correlated with GEDVI (*rho* = 0.93, *P*  = .001).

**Figure 1.**
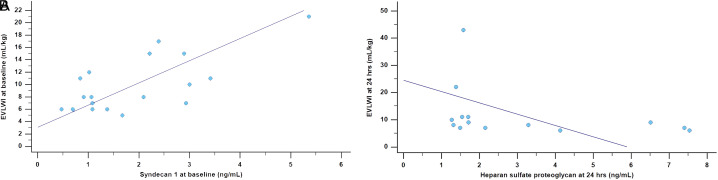
The relationship of glycocalix with severity of pulmonary edema during the study. (A) Correlation of extravascular lung water index (EVLWI) with syndecan 1 concentration at baseline. (B) Correlation of EVLWI with heparan sulfate proteoglycan at 24 hrs after fluid load.

Figure 1 shows the relationship of EG with severity of pulmonary edema. The plasma concentration of S1 correlated positively with EVLWI at baseline (*rho* = 0.48, *P*  = .04), whereas HSPG associated with EVLWI negatively at 24 hours after FLT (*rho* = −0.56, *P*  = .03). In addition, at 24 hours after FLT, the values of HSPG correlated with PPV (*rho* = 0.53, *P*  = .04). 

**Figure 2.**
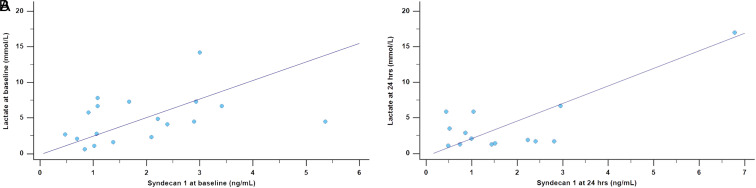
Correlation of lactate concentration with syndecan 1 during the study. (A) Correlation of lactate with syndecan 1 concentration at baseline. (B) Correlation of lactate with syndecan 1 concentration at 24 hrs after fluid load.

Figure 2 demonstrates that the plasma concentration of S1 before FLT correlated with lactate at baseline (*rho* = 0.51,* P*  = .02). Similar association between S1 and lactate was also observed at 24 hours after the FLT (*rho* = 0.76, *P*  = .009).

## Discussion

During our study, we found a relationship of EG components with hemodynamic and metabolic response to fluid therapy in septic shock. The activation of S1 and HSPG was accompanied by increased severity of noncardiogenic pulmonary edema.

In our study, we did not find significant changes in the concentrations of S1 and HSPG during septic shock. The possible explanation could be that at the time of inclusion into the study our patients were already diagnosed with septic shock, thus the multiorgan failure and the activation of the endothelium with glycocalyx damage has been triggered earlier.^6^ The depletion of EG at the onset of sepsis and a decrease in the concentration of its components in plasma in patients with septic shock over time have also been confirmed in a previous study by Ikeda et al.^18^

Although the increase in the plasma concentrations of S1 and HSPG after FLT in our study did not reach statistical significance, Johansson et al.^19^ demonstrated that S1 is a valid marker of EG degradation, and its increment in plasma in patients with septic shock is associated with severe systemic inflammation, coagulopathy, and high mortality. Several other studies showed a significant increase in S1 in the group of patients with septic shock compared with healthy volunteers; however, the authors did not reveal a correlation between the concentration of S1 and mortality.^20-22^ In the work comparing patients with sepsis and after major abdominal interventions, the investigators found that in septic shock, the concentrations of S1 were increased significantly and rose in parallel with markers of inflammation.^23^ Moreover, Ostrowski et al.^24^ observed the relationship between the concentration of S1 and the severity of organ dysfunction in sepsis. Another component of EG, HSPG is also elevated in septic shock compared with patients after neurosurgical interventions; moreover, in patients who died within 90 days the plasma level of HSPG was 4-fold higher than in the group of survivors.^22^

However, the response of EG to infection can differ significantly. Thus, in the study of healthy volunteers who underwent endotoxemia, no significant increase in plasma S1 was obtained either after 4 hours or 6 hours from the beginning of infusion of *Escherichia coli* lipopolysaccharide (LPS), while administration of LPS was accompanied by an early decrease in the level of protein C. Recent studies have demonstrated the heterogeneity of HSPG levels depending on the pathogen strain. In particular, Gram-negative microorganisms lead to higher plasma HSPG concentrations in patients with septic shock.^25^

Only 56% of patients with septic shock in our study were fluid responders, thus further fluid resuscitation was not necessary in almost half of them. In responders, we observed attenuation of tachycardia and an increase in GEDVI at 2 hours after fluid bolus. Although most effects of fluid load were transient, and there were no significant differences in studied parameters between responders and nonresponders, we found relationship between concentration of S1 and global end-diastolic volume in the normovolemic group. By contrast, fluid therapy in patients with decreased GEDVI was not accompanied by EG shedding. Indeed, in sepsis, the fluid therapy restores volume status but, if excessive, can lead to additional damage to the EG and promote capillary leak syndrome.^26-30^ This assumption is confirmed by increased EVLWI and pulmonary vascular permeability in our study. It has been shown that too liberal infusion in septic shock causes degradation of glycocalyx and results in deterioration of its barrier function.^21-24^ In opposite, fluid restriction aiming at prevention of hypervolemia can protect EG by attenuating the release of atrial natriuretic peptide, which induces glycocalyx digestion mediated by matrix metalloproteinase.^29^

In our study, we found an association of S1 and HSPG in septic shock with hemodynamic parameters reflecting both volume status and fluid responsiveness such as GEDVI and PPV. During sepsis, several mediators including endotoxin and cytokines lead to activation of endothelial cells and their structural changes, which in turn can induce the synthesis of endothelial nitric oxide and cause vasoplegia. Degradation of heparan sulfate and hyaluronic acid also results in dilatation of the vascular bed in vivo. Thus, a positive correlation between HSPG and PPV at 24 hours after fluid load can be explained by relative hypovolemia in parallel with systemic vasodilation and endothelial damage that occurs frequently in hyperdynamic septic shock.^12^ During our study, the hyperdynamic state and vasoplegia were observed in nonresponders to fluid load; in parallel, they demonstrated a transient decrease in PPV. However, the value of PPV and SVV in septic shock is limited since these indexes of fluid responsiveness depend on vasopressor dose, presence of spontaneous breathing, right-heart failure, increased intra-abdominal pressure, and other factors.^5^ Therefore, in addition to dynamic parameters of preload, it is important to guide the fluid therapy of shock considering a complex of variables including clinical signs, lactate, arterial and venous blood gases, and volumetric monitoring.

One of the main findings of our study is relationship between components of EG and severity of sepsis-induced pulmonary edema that is confirmed by positive association of S1 plasma concentration and extravascular lung water content before fluid load. Indeed, glycocalyx is a regulator of barrier integrity in the alveolar endothelium and can influence the accumulation of lung fluid.^26^ This is confirmed by last studies in which authors have shown a higher plasma concentration of S1 in patients with septic ARDS, predominantly of extrapulmonary origin,^1,11,15^ like in the case of patients from our study.

Interestingly, in our study, HSPG was associated with EVLWI negatively at 24 hours after FLT. This finding contrasts with studies where authors have demonstrated in the experimental model of sepsis-induced acute lung injury that the diffuse damage to the alveoli is related with increased plasma HSPG concentration.^9^ The possible explanation of this discrepancy can be depletion of HSPG during the clinical course and fluid therapy of septic shock. 

In addition, we observed that the concentration of S1 in patients with septic shock correlates with plasma lactate both before and at 24 hours after fluid load. This finding is consistent with the results of Ikeda et al.^18^ and can be explained by the effects of oxidative stress and tissue hypoperfusion on the endothelium during inflammation. In this scenario, the damage to the glycocalyx occurs, and the normal functioning of the microvasculature is disrupted, which directly affects tissue oxygenation and lactate clearance. On the other hand, according to the modern concept of shock-induced endotheliopathy (SHINE), glycocalyx discharge can serve as a protective mechanism (in particular, during early phase of sepsis), preventing the severity of metabolic response.^10^

It is noteworthy that during sepsis, hypoperfusion, and multiple organ failure further damage to the EG may occur. Although we have not studied the relationship of EG with clinical outcomes, several authors have shown a positive association of plasma S1 level with doses of vasopressors, the volume of fluid therapy, incidence of coagulopathy, hepatic and renal dysfunction, as well as with the duration of ICU stay and mortality.^16,19^ In addition, damage to the EG and development of pulmonary edema during excessive fluid therapy lead to a prolongation of mechanical ventilation, which increases the duration of hospital stay.

The limitations of our study include observational design, small sample size, and a relatively short period of observation with transient effects of fluid load. However, despite these limitations, our findings can be helpful for further work discovering the relationship of EG and mechanisms of cardiopulmonary dysfunction in sepsis.

## Conclusions

The initial state of EG and shedding of its components (HSPG and S1) in septic shock are interrelated with hemodynamic and metabolic disorders. After fluid load in septic shock, the accumulation of extravascular lung water during capillary leak syndrome is associated with degradation of EG components.

### Declaration of Interests:

The authors have no conflict of interest to declare.

## Figures and Tables

**Table 1.** The Clinical Characteristics of the Patients

Parameter	Value
Age (years)	55 ± 16
Gender (male/female)	11/7
Fluid responders/nonresponders	10/8
Fluid balance in 24 hours (mL)	3560 ± 1870
SOFA (baseline)	11 (8-12)
SOFA (24 hours)	9 (7-10)
Dose of epinephrine (μg kg min)	0.37 (0.20-0.50)
Dose of norepinephrine (μg kg min)	0.88 (0.60-1.49)
Diagnosis, n (%)	
Pneumonia	6 (33)
Necrotizing pancreatitis	5 (28)
Peritonitis	6 (33)
Liver abscess	1 (6)
Duration of ICU stay (days)	10 (6-26)
Duration of hospital stay (days)	17 (7-32)
Mortality at day 28, n (%)	8 (44)

ICU, intensive care unit.

**Table 2.** The Changes of Study Parameters

Parameter	Stage		
Baseline	2 hours After FLT	24 hour After FLT
MAP (mm Hg)	81 (65-97)	79 (63-95)	81 (61-101)
CI (L min−1 m−2)	3.23 (2.64-4.29)	2.95 (2.63-3.90)	3.79 (3.21-5.22)^*^
SVRI (dyn × s cm−5 m−2)	1853 (1130-2576)	1770 (1178-2362)	1597(624-2570)
GEDVI (mL m−2)	654 (595-808)	674 (614-743)	724 (559-852)
PPV (%)	16 (9-23)	13 (6-20)	15 (8-22)
SVV (%)	22 (14-30)	15 (9-21)	14 (8-20)
EVLWI (mL kg−1)	7 (5-21)	8 (6-16)	9 (6-23)
PVPI	4.2 (3.1-6.4)	4.5 (3.2-6.5)	5.5 (3.3-6.9)^*^
PaO_2/_FiO_2_ (mm Hg)	228 (177-310)	219 (172-254)	258 (141-348)
Glucose (mmol L−1)	13.1 (9.2-16.7)	13.1 (8.9-17.0)	9.1 (7.7-10.9)
Lactate (mmol L−1)	4.5 (2.7-6.7)	3.7 (2.2-5.8)	2.0 (1.5-4.1)
HSPG (ng mL−1)	2.73 (1.7-7.28)	3.26 (1.87-5.83)	1.70 (1.36-5.58)
S1 (ng mL−1)	1.08 (0.80-2.64)	1.49 (0.87-4.45)	0.93 (0.47-2.27)

^*^ *P* < .05 compared with baseline, Wilcoxon test. CI, cardiac index; EVLWI, extravascular lung water index; GEDVI, global end-diastolic volume index; HSPG, heparan sulfate proteoglycan; MAP, mean arterial pressure; PPV, pulse pressure variation; PVPI, pulmonary vascular permeability index; S1, syndecan 1; SVV, stroke volume variation; SVRI, systemic vascular resistance index.

**Table 3.** The Changes of Study Parameters in Groups of Responders and Nonresponders to Fluid Load

Parameter	Responders (n = 10)			Nonresponders (n = 8)		
Baseline	2 hours After FLT	24 hours After FLT	Baseline	2 hours After FLT	24 hours After FLT
MAP (mm Hg)	84 (74-89)	78 (58-95)	97 (66-108)	85 (81-88)	84 (76-86)	74 (68-78)^*^
HR (bpm)	116 (103-128)	100 (88-115)^*^	109 (83-113)	127 (97-135)	111 (90-124)	104 (97-120)
CI (L min−1 m−2)	2.9 (2.1-3.9)	2.9 (3.4-3.7)	3.4 (2.3-5.3)	3.2 (3.2-6.0)	3.1 (2.6-4.3)	4.0 (3.2-5.3)^*^
SVRI (dyn × s cm−5 m−2)	1855 (1134-3045)	1586 (1302-2247)	1272 (1188-2577)	1446 (961-1942)	1775 (1217-2194)	1081 (731-1661)^*^
GEDVI (mL m−2)	642 (580-687)	668 (604-706)^*^	675 (534-892)	771 (636-840)	717 (640-764)	713 (519-833)
PPV (%)	19 (9-20)	12 (7-21)	17 (8-22)	16 (12-19)	8 (7-16)^*^	14 (12-16)
SVV (%)	24 (9-28)	14 (10-17)	15 (9-23)	25 (17-32)	13 (7-18)	12 (9-16)
EVLWI (mL kg−1)	8 (6-11)	9 (6-12)	10 (7-13)^*^	15 (7-16)	9 (7-12)	8 (6-11)
Lactate (mmol L−1)	2.8 (2.1-7.1)	2.4 (2.2-6.8)	2.6 (1.3-4.1)	4.5 (3.6-6.6)	4.7 (4.7-5.3)	1.9 (1.6-5.5)
Dose of norepinephrine (mcg kg min)	0.73 (0.60-1.47)	0.50 (0.24-0.90)	0.05 (0.01-0.72)^*^	0.82 (0.21-1.05)	0.57 (0.10-1.0)	0.08 (0.02-0.43)
Duration of ICU stay (days)	9 (6-24)			10 (8-26)		
Duration of hospital stay (days)	16 (8-32)			15 (7-26)		
Mortality at day 28 (n [%])	3 (33%)			5 (63%)		

^*^ *P* < .05 compared with baseline, Wilcoxon test. CI, cardiac index; EVLWI, extravascular lung water index; GEDVI, global end-diastolic volume index; HR, heart rate; MAP, mean arterial pressure; PPV, pulse pressure variation; SVV, stroke volume variation; SVRI, systemic vascular resistance index.
